# Evaluating The Glasgow Blatchford Score for Upper Gastrointestinal Bleeding Risk Stratification in A Community Hospital: A Retrospective Study.

**DOI:** 10.51894/001c.137546

**Published:** 2025-05-01

**Authors:** Hind H. Neamah, Alexandra Davies, Anthony Teta, Grace D. Brannan, Sami Abdelaziz, Bruce Kovan

**Affiliations:** 1 Department of Internal Medicine, Mount Clemens, MI, USA McLaren Health Care- Macomb Hospital; 2 Internal Medicine, East Lansing, MI, USA Michigan State University, College of Osteopathic Medicine; 3 Gastroenterology, East Lansing, MI, USA Michigan State University, College of Osteopathic Medicine,; 4 Department of Gastroenterology, Lansing, MI, USA McLaren Health Care- Greater Lansing Hospital; 5 Graduate Medical Education, Mount Clemens, MI, USA McLaren Health Care- Macomb Hospital; 6 GDB Research and Statistical Consulting, Athens, OH, USA; 7 College of Osteopathic Medicine, East Lansing, MI, USA Michigan State University; 8 Department of Gastroenterology, Mount Clemens, MI, USA McLaren Health Care- Macomb Hospital; 9 College of Osteopathic Medicine, Gastroenterology, East Lansing, MI, USA Michigan State University

**Keywords:** Upper gastrointestinal bleed (UGIB), Glasgow Blatchford Score (GBS), Hemostatic Intervention, gastroenterology

## Abstract

**INTRODUCTION:**

Upper gastrointestinal bleeding (UGIB) is the most common emergency in gastroenterology. The Glasgow Blatchford Score (GBS) is a validated tool used for risk stratification. The cutoff values for GBS to predict the need for clinical intervention, endoscopic treatment, and mortality, are not consistent. To determine the relationship between mean GBS score and the need for hemostatic intervention, and blood transfusion, and to evaluate quality of care and proper allocation of resources at our midwestern community hospital.

**METHODS:**

In this cross-sectional study, we retrospectively extracted records for patients ≥18 years who were admitted for UGIB and underwent esophagogastroduodenoscopy between July 2018 and July 2020. GBS was calculated for each observation. Multivariate analysis and a logistic regression model were performed to predict the GBS score, and the odds ratio, associated with the need for hemostatic intervention and blood transfusion while controlling for confounding factors.

**RESULTS:**

The GBS sample mean score was 11.17. Those who required hemostatic intervention and blood transfusion scored significantly higher GBS (13.18 versus 10.79) and (13.57 versus 9.21), respectively. A GBS of >10 was associated with higher odds at 21.84 (CI: 10.324,46.185, P<0.001) and 5.085 (CI: 1.864, 13.872, P=0.001) for receiving blood transfusion and hemostatic intervention, respectively. A cutoff of 10 was 22.41% sensitive and 95.41% specific for requiring hemostatic interventions and 66.67% sensitive and 89.91% specific for receiving blood transfusion.

**CONCLUSION:**

There is a clinical role to using the GBS even at a score higher than 2 to further stratify the severity of UGIB and determine the need for intervention. The sensitivity of a score of 10 on the GBS in this dataset was low. A cutoff with higher sensitivity is needed to stratify a life-threatening condition such as UGIB.

## INTRODUCTION

Upper gastrointestinal bleeding (UGIB), a life-threatening condition, is the most common emergency in gastroenterology.^1^ At an incidence rate of 48-160/100,000 it accounts for 300,000 hospitalizations per year and a healthcare cost of $7.6 billion in the United States as estimated in the year 2009.^1^ Not all patients with UGIB require intervention in the hospital, in fact 25% could be discharged home safely.^2–4^ Therefore, the American College of Gastroenterology (ACG)^5^ and The International Consensus Guidelines^6^ recommend applying tools for risk stratification to guide medical decisions.

The Glasgow Blatchford Score (GBS) uses a set of non-endoscopic criteria including; blood urea, hemoglobin level, systolic blood pressure, pulse rate, presence of melena, presence of syncope, and history of hepatic disease and heart failure to stratify UGIB risk.^7^ Although the predictive value of GBS has been extensively validated, proving that it does correlate with the need for treatment in UGIB, its clinical utility could be improved. For example, it can be used to predict the need for clinical intervention,^2–4^ endoscopic treatment, and mortality,^7–9^ however, the cutoff values are not consistent.^10,11^ Additionally, the ACG guidelines for the management of non-variceal bleeding recommend safe outpatient management at a cutoff of ≤1 on the GBS, however, the data for supporting this cutoff as evaluated by the GRADE system is weak.^12^ Therefore, the ACG calls for further studies to investigate the risk assessment tools which we currently use to improve the management of patients with UGIB.^12^ We seek to add to the evidence by further defining the clinical significance of GBS.

### Purpose of study

Our primary objective was to determine the relationship between mean GBS score and receiving hemostatic intervention, and blood transfusion at our midwestern community hospital. Our secondary objective was to evaluate the quality of care according to guidelines at our hospital and the proper allocation of resources.

## METHODS

Under The McLaren Macomb Institutional Review Board (IRB) approval (IRB 2020-00051) and oversight for this cross-sectional study, we extracted data from electronic medical records retrospectively for patients 18 years or older, who were admitted to McLaren Macomb Hospital with any type of UGIB and underwent esophagogastroduodenoscopy (EGD) between 7/1/2018 - 7/31/2020. The patients’ encounters of interest were identified using procedure codes for EGD as defined by the tenth edition of the International Classification of Disease (ICD 10) including 06L38CZ, 0D538ZZ, 0D568ZZ, 0D578ZZ, 0D598ZZ, 0DB18ZX, 0DB28ZX, 0DB48ZX, 0DB58ZX, 0DB68ZX, 0DB78ZX, 0DB98ZX, 0DBA8ZX, 0DJ08ZZ.^13^ Data was extracted using procedure codes, rather than ICD-10 diagnostic codes corresponding to UGIB, because of our interest in evaluating the quality of care and proper allocation of resources at our hospital. ACG guidelines recommend safe outpatient management for those who score ≤1 on the GBS. A guideline- based approach would mean that the percentage of those undergoing endoscopy with a GBS score of ≤1 should be kept at a minimum. Patients who were less than 18 years of age who were initially admitted for other reasons and developed UGIB during their hospital stay, (or those) readmitted for UGIB within three months of their initial presentation were excluded from the study.

We extracted gender, age, history of liver disease, other comorbidities (defined as any personal history of any chronic disease), history of gastrointestinal malignancy, diabetes, bleeding disorder, use of non-steroidal anti-inflammatory medications within the last three months, current use of any antiplatelet agents including aspirin, clopidogrel, and ticagrelor, and current use of blood thinners including apixaban, dabigatran, edoxaban, rivaroxaban, warfarin, bivalirudin, argatroban, and heparin. We also extracted the nine variables that are necessary for calculating GBS scores as assessed at admission including Blood urea nitrogen (BUN), hemoglobin levels for men, hemoglobin levels for women, systolic blood pressure, pulse rate, presentation with syncope, presentation with melena, having a diagnosis of heart failure, and having a diagnosis of liver failure. The endoscopy report for each clinical encounter was reviewed for the need for surgical intervention and hemostatic interventions. Hemostatic interventions were defined as endoscopic interventions (such as banding, epinephrine injection, Hemospray© use, cautery, and clip placement) and advanced radiological interventions in the form of arterial embolization. Each of the variables was coded as a categorical variable (yes/no). Charts were also assessed for receiving blood transfusion as a categorical variable (yes/no).

*Calculating GBS:* The GBS was calculated as a continuous variable for each patient by tallying the nine categorical variables that comprise the score. Each of those nine variables was assigned a value ranging from 0-6 according to the GBS calculation.^2,14^ Total score was generated by tallying the categories for each patient and a total sample mean was generated.

We generated descriptive statistics such as frequencies, percentages, and means. We evaluated the mean GBS score per category via an Independent T-test and t, standard deviation (SD), and range. We performed a multivariable linear regression analysis to predict the GBS score that is associated with the need for hemostatic intervention and the need for blood transfusion while controlling for age (>60 versus <60), gender (female versus male), presence of GI malignancy (yes/no), presence of liver disease (yes/no), use of antiplatelets (yes/no), use of blood thinners (yes/no), and use of NSAID within the past three months (yes/no). We then converted the continuous GBS score into a categorical variable and performed logistic regression using a model controlling for all the variables we used in the linear regression, to determine the odds of patients with GBS score > 10 vs ≤10 needing a hemostatic treatment or blood transfusion. We based our cut-off (10) on the average GBS for those who did not receive blood transfusion and those who did not require hemostatic intervention. We used IBM SPSS version 25 (IBM Corp., Armonk, NY) to analyze the data. Statistical significance was set at a *p ≤ 0.05*. Co-author G. Brannan performed the analysis.

## RESULTS

Two hundred and eighty-three patients met our inclusion criteria and all of them were included in the analysis. The mean age of the study population was 66.67 years old. The GBS mean score was 11.17 and the scores ranged from 3 to 19. Demographic variables of the sample are described in Table 1.

**Table 1.** Frequency distribution of the descriptive characteristics of the study sample and mean GBS score of the study sample

**Variable**	**Frequency**	**Percentage**	**GBS Mean (SD)^ϕ^**	**CI^λ^**	**P**
**Age**					
>=60	201	71.0	11.78 (3.61)	(-3.2 to -1.01)	<.001
< 60	82	29.0	9.67 (4.45)		
**Gender**					
Female	136	48.1	10.86 (3.91)	(-0.34 to 1.52)	.215
Male	147	51.9	11.45 (4.04)		
**Presence of Comorbidity**					
No	23	8.1	9.35 (4.21)	(-3.67 to -0.29)	.022
Yes	260	91.9	11.33 (3.93)		
**Presence of GI malignancy**					
No	271	95.8	11.11 (4.02)	(-3.18 to 0.40)	.117
Yes	12	4.2	12.50 (2.75)		
**Ever Been diagnosed with any malignancy**					
No	235	83.0	11.28 (4.01)	(-0.56 to 1.92)	.284
Yes	48	17.0	10.60 (3.84)		
**Use of Antiplatelets**					
No	191	67.5	11.02 (4.08)	(-1.44 to 0.55)	.378
Yes	92	32.5	11.47 (3.78)		
**Using Dual Antiplatelet**					
No	252	89.0	11.02 (4.03)	(-2.86 to 0.11)	.070
Yes	31	11.0	12.39 (3.37)		
**Use of NSAID**					
No	230	81.3	11.36 (3.93)	(-0.173 to 2.21)	.094
Yes	53	18.7	10.34 (4.13)		
**Using Blood Thinners**					
No	231	81.6	10.99 (4.02)	(-2.15 to 0.25)	.120
Yes	52	18.4	11.94 (3.78)		
**Using Antiplatelets and blood thinners**					
No	260	91.9	11.03 (4.06)	(-2.84 to -0.39)	.011
Yes	23	8.1	12.85 (2.62)		
**History of bleeding disorder**					
No	279	98.6	11.23 (3.97)	(0.82 to 8.65)	.018
Yes	4	1.4	6.50 (1.92)		
**Hepatic disease**					
No	237	83.7	10.83 (3.82)	(-3.30 to -0.82)	.001
Yes	46	16.3	12.99 (4.39)		
**History of HF**					
No	236	83.4	10.68 (4.00)	(-3.95 to -1.93)	<.0001
Yes	47	16.6	13.62 (3.00)		

ϕ SD= standard deviation.

λ CI= confidence interval

The lowest mean GBS score in our sample was 2 denoting that no patients with a GBS of ≤1 received endoscopy, in accordance with ACG guidelines. The mean GBS scores were compared through the Student T-test for each variable (Table 1). Mean GBS was found to be significantly higher among subjects who were older than 60 years of age (11.78 versus (vs) 9.67, confidence interval (CI) -3.2 to -1.01, *p*=0.001) or other comorbidities (11.33 vs 9.35, CI -3.67 to -0.29, *p* = 0.022,). The results were also significant for subjects who used dual antiplatelet therapy in combination with an anticoagulant (12.85 vs 11.02, CI -2.84 to -0.39, *p* = 0.011) and in those who did not have a bleeding disorder (11.23 vs 6.5, CI 0.82 to 8.65, *p* = 0.18). Mean differences in GBS with gender, any use of antiplatelets, use of blood thinners, and use of non-steroidal anti-inflammatory medications were not found to be significant.

Only 44/283 (15.5%) patients required hemostatic intervention including cautery 14 (4.9%), banding 11 (3.9%), clip replacement 2 (0.7%); and 17 patients (6.1%) required a combination of treatments conducted together to control the bleeding.

In the multivariable linear regression analysis, while controlling for all other confounding factors, those who needed hemostatic interventions scored 1.546 (CI: 0.291-2.801, P=0.016) points higher GBS score when compared to those who did not need a hemostatic intervention (mean GBS13.18 vs 10.79, T-test CI: -3.42 to -1.36, p=0.00001). Similarly, those who needed blood transfusion scored 4.296 (mean GBS 13.57 vs 9.21, T-test CI: -5.11 to -3.60, p=0.00001)) points higher than those who did not need a blood transfusion (Table 2).

**Table 2.** Need For Homeostatic Treatment and Blood Transfusion Per GBS^f^

**N**(%)		**Student T-test**		**Multivariate Analysis**					**Logistic Regression**			
		GBS Mean (SD) ^a^	CI^b^	P^c^	B^d^	SE^e^	CI	P	OR^f^	CI	P	
**Need for Hemostatic Treatment during Endoscopy** ^a^												
Yes	44 (15.5%)	13.18 (2.97)	(-3.42, -1.36)	**<.0001**	1.546	0.638	(0.291, 2.801)	**0.016**	5.085	(1.864, 13.872)	**0.001**	
No	239 (84.5%)	10.79 (4.04)										
**Need for blood transfusion**												
Yes	127 (44.9%)	13.57 (2.49)	(-5.11, -3.60)	**<.0001**	4.296	0.368	(3.571, 5.021)	**0.000**	21.836	(10.324,46.185)	**<0.001**	
No	156 (55.1%)	9.21 (3.90)										

^f^Models are controlled for gender, age, presence of gastrointestinal malignancy, current use of antiplatelet agents, current use of blood thinners, use of non-steroidal anti-inflammatory medications with in the past three months, history of liver disease.

^a^SD= standard deviation.

^b^CI= confidence interval.

^c^P value.

^d^B coefficeint of the linear regression model.

^e^Standard error of the linear regression model.

^f^Odds ratio of the logistic regression model for GBS <10 versus <=10.

We performed further analysis to determine the adjusted odds of receiving blood transfusion for GBS >10 using logistic regression and accounting for the same variables we controlled for in the linear regression. We found that patients who scored >10 on GBS are 21.84 (CI: 10.324,46.185, P<0.001) times significantly more likely to receive a blood transfusion and 5.085 (CI: 1.864, 13.872, P=0.001) times significantly more likely to need hemostatic treatment during endoscopy than those who scored ≤10 (Table 2). The cutoff of 10 was 66.67% sensitive and 89.91% specific for blood transfusion and 22.41% sensitive and 95.41% specific for hemostatic interventions.

## DISCUSSION

This study found that a higher mean GBS is associated with a higher likelihood of receiving hemostatic intervention and blood transfusion with a mean difference of 1.546 and 4.296 points and a total mean score of 13.18 and 13.57, respectively. This means that even at a score of ≥1 or ≥2, the mean GBS correlates with the severity of the UGIB, showing that risk stratification could be clinically significant at higher scores. The lowest GBS in our sample was three, which means that no patient with a GBS ≤ 2 received an EGD at our hospital during the period studied. Those who scored ≤ 2 on the GBS were clinically identified as not being sick to the level of necessitating a hemostatic procedure denoting good allocation of resources in this community hospital. This finding agrees with accumulating evidence recommending expanding the cutoff from ≤1 to ≤2.^7,15^ For example, a large retrospective study including 399 patients and a comprehensive metanalysis study of 38 studies that were included from 2153 citations, found that a GBS ≤ 2 maintained a prognostication of low-risk patients comparable to the prognostication suggested by a score of ≤ 1^16^ and a negative predictive value for excluding endoscopy of 98.53% at ≤2 versus 100% at a cutoff of ≤ 1.^15^ These results agree with our findings of lower risk in those who scored GBS ≤ 2. However, one study found that a GBS ≤2 had an associated 30-day combined adverse outcome defined as blood transfusion, intervention, or death of 8% (n=3/37) when patients were discharged directly from the emergency room.^17^ The small sample size of three patients only and the fact that one of the three patients died of respiratory arrest limit the generalization of these findings.^17^

Ninety-one percent of our patients had at least one co-morbidity; this could be explained by the mostly elderly population with a mean age of 66.67 years (Table 1). The likelihood of having at least one co-morbidity increases with age.^13^ Additionally, mean GBS was statistically significantly higher for those who used dual anti-platelets - this was expected because the use of antiplatelet medications can increase the risk of UGIB.^14^ Our sample had a statistically significantly higher GBS in those who did not have a history of bleeding disorder, however, the sample size was small (n=4), which might have skewed the results. NSAID use in the past three months did not seem to increase the GBS score, documented medical evidence showing current use of NSAID is associated with UGIB,^18^ therefore, future research should focus on current rather than historical NSAID use.

We also found a significant difference in average GBS by age group (9.67 in those < 60 years old, versus 11.78 ≥ 60 years old). Other studies examined the relationship of age with UGIB at the cutoff of 60,^19,20^ since patients ≥65 years of age are more likely to have comorbidities deeming them unfit to tolerate EGD, thus limiting their sample size at a higher cut-off.^11^ The percentage of patients who were ≥ 60 was 29% in our study, comparable to other studies ranging from 23%-33%.^19,20^ One study found less frequency of intervention in patients who are ≥ 60 years old, however, it did not control for any confounding factors.^20^ In our sample, increased age was associated with a higher score on the GBS, this finding is likely related to increased comorbidities in this age group, rather than due to an independent relationship to the UGIB.

We found out that a GBS cut-off of 10 for the secondary analysis was reasonable to use since the sample mean as examined by demographic variables ranged between 9-11 for most of those variables as can be seen in Table 1. A GBS of 10 was 22.41% sensitive and 95.41% specific for requiring hemostatic interventions and 66.67% sensitive and 89.91% specific for receiving blood transfusion. Several studies have attempted to expand the clinical utility of GBS by determining a cutoff value associated with increased risk for receiving an intervention or undergoing a blood transfusion. For hemostatic intervention, previous studies set different cutoffs such as 8 (sensitivity=83%, specificity=50%),^11^ ≤ 7 (sensitivity=80.7%,specificity=57.4%),^8^ 15, (sensitivity=39%, specificity=76%).^21^ For blood transfusion, previous studies used the following cutoffs for mean GBS:6.5^22^ (sensitivity= 91%, specificity=85%), 7^11^ (sensitivity=52%, specificity=60%), and 14 (sensitivity=61%, specificity=78%).^21^

In our study, the cutoff was more specific for those who needed hemostatic intervention, and less specific for those who needed blood transfusion. We can conclude that GBS is more specific in determining the need for intervention rather than blood transfusion as the need for blood transfusion follows a different set of guidelines such as surviving sepsis guidelines and clinical judgment. This is a comment regarding specificity and not the sensitivity of GBS in predicting hemostatic interventions when compared to the need for blood transfusion and not to disagree with the literature that validates the use of GBS in predicting need for blood transfusion, as the sensitivity for such prediction is still high.

The sensitivity for the cutoff of 10 was low for hemostatic intervention. A GBS score <10 fails to identify some of those who would need hemostatic intervention. In this illness, which is associated with high morbidity and mortality, we argue that a cutoff of reasonable sensitivity is needed. GBS is more accurately validated in patients who have non-variceal UGIB and is used in those patients per the ACG guidelines.^2,9^ Our sample included both variceal and non-variceal bleeding. We recommend that different GBS cutoffs should be studied in patients with non-variceal UGIB than those with variceal UGIB. Although at presentation, the reason for the UGIB may be unknown, there is clinical relevance to this separation especially since most variceal UGIB are presented with catastrophic bleeding and will likely need EGD. Patients with variceal bleeding, who by disease process have liver disease, would automatically score a GBS of 2,^15^ and such patients in the correct clinical scenario and a history of a previous variceal bleed will get an EGD regardless of their GBS even in the absence of catastrophic UGIB. This is mainly to reduce interference in studies that seek to determine the mean GBS score when the clinical diagnosis is uncertain and the need for endoscopy is unclear, such as in non-variceal bleeding.

### Study Limitations

This cross-sectional study was performed in one community hospital and therefore the generalizability of the results is limited. We did include all types of UGIB in this study and we did not stratify the analysis per variceal versus non-variceal UGIB. Stratifying by the diagnosis of bleeding might produce more clinically significant results when it comes to the cutoff per the ACG guidelines. We retrospectively included any UGIB and extracted our sample by EGD procedure codes and did not include the final diagnosis (variceal vs non-variceal bleeding). This may limit the generalizability of our study findings as GBS is more accurate in patients with non-variceal bleed. We extracted data retrospectively based on ICD10 codes for those who underwent EGD because of our primary goal in assessing the quality of care in our hospital for patients with UGIB. Prospective data collection may yield more information about patients who did not undergo endoscopy and their final outcomes. Current use of NSAIDs should have been studied rather than historic use of NSAID (over the past three months), although this includes those who used NSAIDs at the time of the hospitalization, it leads to over-representation of those who are not at immediate risk of UGIB due to historic NSAID use.

## CONCLUSIONS

The quality of care for patients with UGIB in our hospital is in accordance with the clinical guidelines for management of UGIB indicating the proper allocation of resources per clinical guidelines for EGD. There is a clinical role to using the GBS even at a score higher than 2 to further stratify severity of UGIB and determine the need for intervention. The sensitivity of a score of 10 on the GBS in this dataset was low. A cutoff with higher sensitivity is needed to stratify a life-threatening condition such as UGIB. Future research should focus on studying GBS cutoffs in non-variceal UGIB separately from variceal UGIB.

### Conflicts of interest

The authors report no financial support or conflicts of interest in the completion of this project and paper.
